# THE IMPACT OF BEHAVIORAL LIFESTYLE INTERVENTION ON INFLAMMATORY CYTOKINES IN OLDER ADULTS WITH TYPE 2 DIABETES

**DOI:** 10.1093/geroni/igac059.2172

**Published:** 2022-12-20

**Authors:** Rozmin Jiwani, Monica Serra, Sara Espinoza, Darpan Patel

**Affiliations:** UTHSCSA, San Antonio, Texas, United States; UT Health San Antonio, San Antonio, Texas, United States; Sam and Ann Barshop Institute, UT Health San Antonio, San Antonio, Texas, United States; UT Health San Antonio, San Antonio, Texas, United States

## Abstract

Chronic inflammation, a hallmark of type 2 diabetes (T2D) and adiposity, increases the risk for age-related co-morbidities, including physical frailty. This study aimed to examine the effects of a mobile technology-enhanced behavioral lifestyle educational intervention on frailty and associated clinical, inflammatory, and laboratory outcomes in overweight older adults with T2D. Twenty participants (≥65 years, BMI ≥25 kg/m2) with T2D were recruited to complete a single-arm 6-month lifestyle intervention modified from the Action for Health in Diabetes (Look AHEAD) study, enhanced with technology for self-monitoring of diet and physical activity. Clinical assessments were collected at baseline and end of the study and analyzed with paired t-tests. Inflammatory cytokines (interleukin [IL]2, IL4, IL6, IL8, IL10, granulocyte-macrophage colony-stimulating-factor [GM-CSF], interferon [IFNγ], tumor necrosis factor [TNFα]) were quantified using a commercially available multiplex kit (Millipore Sigma, Burlington, MA, USA). Clinical lab analysis was performed by Quest Diagnostics (Dallas, TX, USA). Eighteen participants completed the study (71.5±5.3 years; 56% female; 50% Hispanic). At baseline, 13 participants were pre-frail, 4 were frail based on Fried frailty criteria. Inflammatory cytokines and liver enzymes values were within normal limits. At follow-up, the following outcomes significantly improved: frailty score -44% (p=0.01), BMI -3% (P< 0.00), alanine transaminase -18% (p=0.03), aspartate aminotransferase -13% (p< 0.00), IL2 -33% (p=0.01), IL4 -31% (p=0.05), IFNγ -36% (p=0.03), GM-CSF -43% (p=0.03). No other significant differences were observed. The results suggest the efficacy of a technology-enhanced lifestyle intervention on frailty and associated clinical, inflammatory, and laboratory outcomes in overweight older adults with T2D.

